# Ventricular strain analysis in patients with no structural heart disease using a vendor-independent speckle-tracking software

**DOI:** 10.1186/s12872-020-01559-1

**Published:** 2020-06-05

**Authors:** Hongmei Xia, Darwin F. Yeung, Cristina Di Stefano, Stephen S. Cha, Patricia A. Pellikka, Zi Ye, Jeremy J. Thaden, Hector R. Villarraga

**Affiliations:** 1 Department of Ultrasound, Xinqiao Hospital, Third Military Medical University, Chongqing, China; 2 Department of Cardiovascular Diseases, Mayo Clinic, Rochester, Minnesota, 200 First St SW, Rochester, MN 55905 USA; 3 Hypertension Unit, Department of Medical Sciences, University of Torino, Torino, Italy; 4 Division of Biomedical Statistics and Informatics, Mayo Clinic, Rochester, MN USA

**Keywords:** 2D speckle-tracking echocardiography, Strain, Myocardial mechanics

## Abstract

**Background:**

Ventricular strain measurements vary depending on cardiac chamber (left ventricle [LV] or right ventricle [RV]), type of strain (longitudinal, circumferential, or radial), ventricular level (basal, mid, or apical), myocardial layer (endocardial or epicardial), and software used for analysis, among other demographic factors such as age and gender. Here, we present an analysis of ventricular strain taking all of these variables into account in a cohort of patients with no structural heart disease using a vendor-independent speckle-tracking software.

**Methods:**

LV and RV full-thickness strain parameters were retrospectively measured in 102 patients (mean age 39 ± 15 years; 62% female). Within this cohort, we performed further layer-specific strain analysis in 20 subjects. Data were analyzed for global and segmental systolic strain, systolic strain rate, early diastolic strain rate, and their respective time-to-peak values.

**Results:**

Mean LV global longitudinal, circumferential, and radial strain values for the entire cohort were − 18.4 ± 2.0%, − 22.1 ± 4.1%, and 43.9 ± 12.1% respectively, while mean RV global and free wall longitudinal strain values were − 24.2 ± 3.9% and − 26.1 ± 5.2% respectively. Women on average demonstrated higher longitudinal and circumferential strain and strain rate than men, and longer corresponding time-to-peak values. Longitudinal strain measurements were highest at the apex compared with the mid ventricle and base, and in the endocardium compared with the epicardium. Longitudinal strain was the most reproducible measure, followed closely by circumferential strain, while radial strain showed suboptimal reproducibility.

**Conclusions:**

We present an analysis of ventricular strain in patients with no structural heart disease using a vendor-independent speckle-tracking software.

## Background

Strain and strain rate are sensitive measures of myocardial function that allow early detection of systolic dysfunction even before changes in ejection fraction (EF) [1–6]. However, strain measurements vary depending on cardiac chamber (left ventricle [LV] or right ventricle [RV]), type of strain (longitudinal, circumferential, or radial), ventricular level (basal, mid, or apical), myocardial layer (endocardial or epicardial), software used for analysis, and demographic factors such as age and gender [7–18].

Previous studies have reported reference values for strain, taking some but not all of these parameters into account [7, 10–13, 16–21]. Furthermore, many of these studies were performed in healthy subjects and therefore do not include the population of patients we encounter in practice who are referred for echocardiography for a clinical indication.

Here, we present an analysis of ventricular strain using a vendor-independent speckle-tracking software in a single cohort of patients who were referred for echocardiography but were found to have no overt evidence of structural heart disease.

## Methods

### Study population

We retrospectively screened 106 patients who were identified as having “normal echocardiograms” in our institutional echocardiography database between April 2009 and January 2014. The echocardiograms screened therefore represented a convenience sample of studies in which the interpreting cardiologist deliberately labeled the study “normal” to draw attention to the lack of any identifiable abnormality found using conventional echocardiographic parameters. These patients would therefore demonstrate the following based on the American Society of Echocardiography guidelines: normal ventricular size, wall thickness, and function including normal LV diastolic function; absence of ventricular wall motion abnormalities; estimated right ventricular systolic pressure within normal limits; no evidence of valvular stenosis; no more than physiologic regurgitation; normal-sized great vessels; no evidence of constriction; no more than physiologic pericardial effusion; and no evidence of congenital heart disease [22–26]. Exclusion criteria included a history of cardiovascular disease, evidence of cardiomyopathy on echocardiogram, non-sinus rhythm, or suboptimal 2D imaging quality.

Patients were referred for echocardiography for a variety of clinical indications, which fall under three broad categories: Group 1 patients were those who were referred for cardiac symptoms (chest pain, dyspnea, palpitations, or syncope), an abnormal electrocardiogram, or for a baseline evaluation prior to chemotherapy initiation; Group 2 patients were those who had a family history of cardiomyopathy including hypertrophic cardiomyopathy, cardiac amyloidosis, idiopathic dilated cardiomyopathy, or non-compaction cardiomyopathy; and Group 3 patients were those who had systemic conditions or prior exposures in which screening for cardiomyopathy is recommended including systemic amyloidosis, hypereosinophilia, muscular dystrophy or suspected mitochondrial disorders, or prior chemotherapy or radiation therapy (Supplementary Table 1).

### Image acquisition and strain analysis

**Fig. 1:**
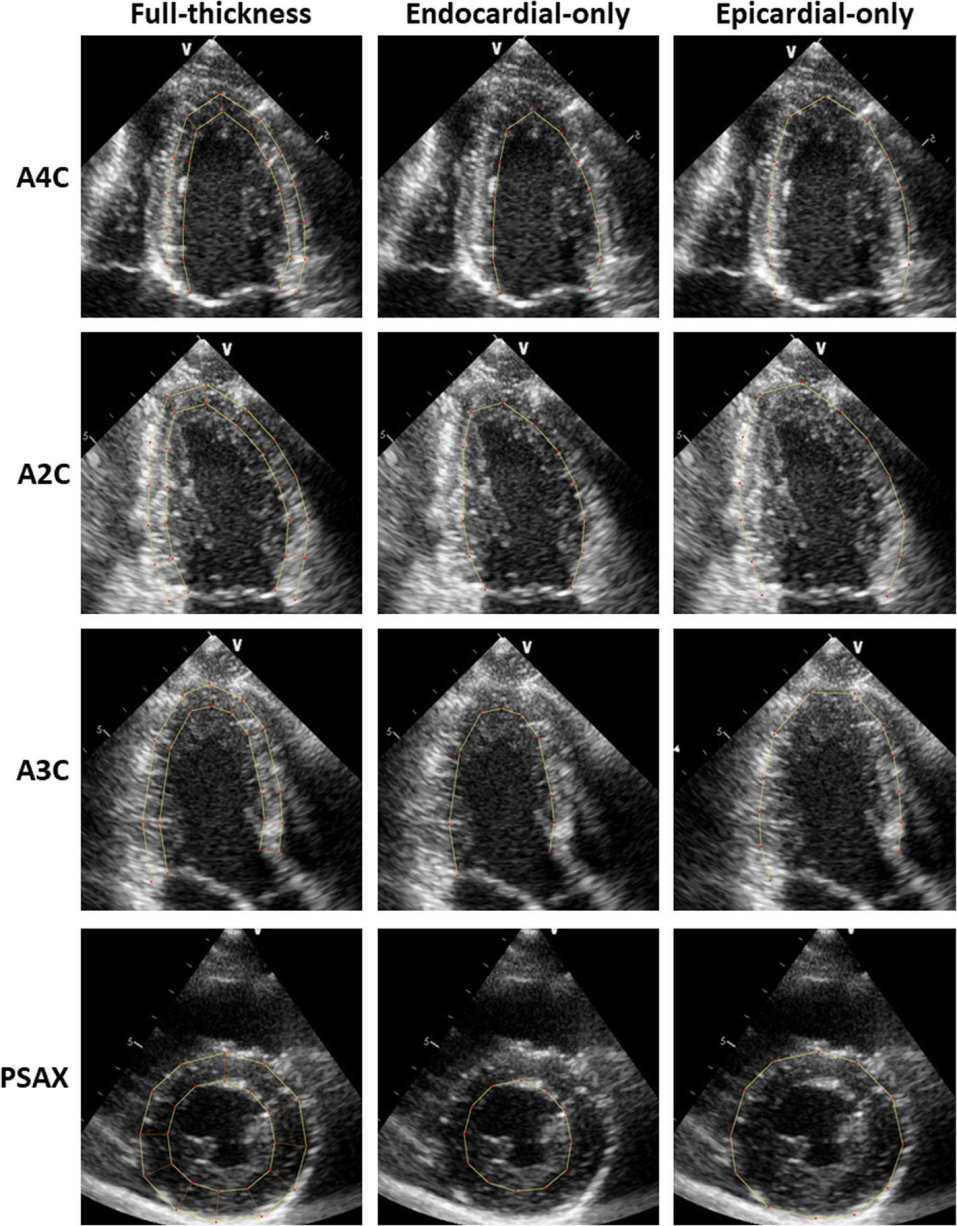
Layer-specific strain analysis of the left ventricle. Longitudinal strain measurements were obtained from the apical 4-chamber (A4C), 2-chamber (A2C), and 3-chamber (A3C) views. Circumferential and radial strain measurements were obtained from the parasternal short-axis (PSAX) view at the papillary muscle level. Full-thickness measurements were performed by tracing an inner border at the endocardium and an outer border at the epicardium. Endocardial-only strain measurements were performed using the same inner endocardial border as the full-thickness measurements. Epicardial-only measurements were performed by approximating the same outer epicardial border as the full-thickness measurements

**Fig. 2:**
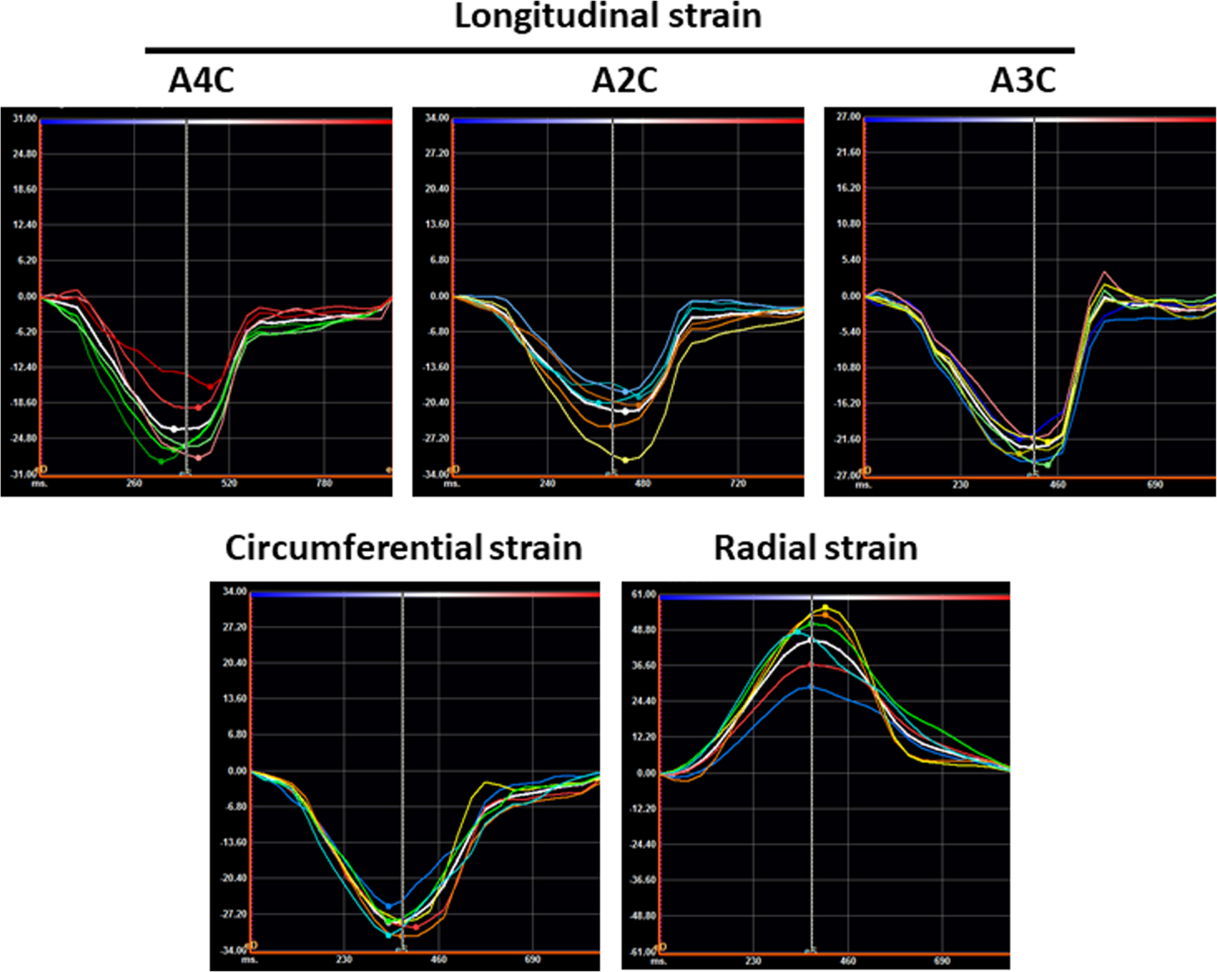
Left ventricular strain curves. Average and segmental longitudinal strain curves were obtained from the apical 4-chamber (A4C), 2-chamber (A2C), and 3-chamber (A3C) views. Average and segmental circumferential and radial strain curves were obtained from the parasternal long-axis view at the level of the papillary muscle. Global longitudinal and circumferential strain measurements were derived from the nadir of the average longitudinal and circumferential strain curves respectively. Global radial strain measurements were derived from the peak of the average radial strain curve

All patients underwent a transthoracic echocardiographic examination with a standard commercially available ultrasound system [Vivid 7, General Electric (GE) Medical Systems, Fairfield, Connecticut] and a 1.5–4.3 MHz M4S transducer. Mean frame rates were 54.4 ± 10.9 Hz for grayscale imaging. Strain measurements were performed offline with the vendor-independent 2D Cardiac Performance Analysis speckle-tracking software (Image Arena version 4.6, TomTec Imaging Systems, Unterschleissheim, Germany) from archived studies in Digital Imaging and Communications in Medicine (DICOM) format (Figs. 1 and 2).

Full-thickness strain measurements were performed for all patients in the cohort. The points in the cardiac cycle in which the ventricles reached maximum and minimum volumes represented end-diastole and end-systole respectively. LV longitudinal strain parameters were measured from the apical 4-chamber, 2-chamber, and 3-chamber views (Fig. 1). Circumferential and radial strain parameters were measured from the parasternal short-axis view at the mid-papillary level (Figure 2) as described in previous studies [12, 16, 17]. The LV was divided into six segments in each view. Full-thickness LV global longitudinal, circumferential, and radial systolic strain measurements were further compared between men and women, and between pre-defined age categories (< 40, 40–59, or ≥ 60 years of age).

RV longitudinal strain parameters were measured from the modified RV-focused apical 4-chamber view. Global RV longitudinal strain was evaluated by averaging peak strain values of six segments: three from the RV free wall and three from the RV septum. RV free wall longitudinal strain was averaged from the three free wall segments only.

Measurements for each subject included global and segmental myocardial strain parameters, including peak systolic strain, peak systolic strain rate, peak early diastolic strain rate, and their respective time-to-peak values. Global longitudinal, circumferential, and radial strain parameters were calculated by averaging the peak strain values.

In addition to full-thickness strain analysis, we performed layer-specific strain analysis in 20 randomly selected patients within the cohort. Three layer-specific measurements were performed per subject: full-thickness, endocardial-only, and epicardial-only (Figures 1 and 2). The endocardial tracking boundary remained the same for the full-thickness and endocardial-only measurements. Analysis was performed in the same cardiac cycle for each of the layer-specific measurements.

### Reproducibility

The global full-thickness strain measurements of the 20 patients who underwent layer-specific strain analyses were repeated by the same investigator (H.X.) several months after the initial analysis to determine intra-observer variability and performed by a second investigator (Z.Y.) to assess inter-observer variability.

### Statistical analysis

Data were summarized as mean ± standard deviation. Deformation parameters were compared between the different myocardial layers using one-way repeated measures analysis of variance (ANOVA). A *P* value < 0.05 was considered statistically significant. When the *P* value was statistically significant, the paired *t* test was used to identify the pairwise difference. Differences between subgroups based on age were assessed with the Tukey-Kramer honestly significant difference test and between subgroups based on sex with the Student *t* test. Shapiro-Wilks test was chosen for testing the normality of the data. Intra-class correlation coefficients (ICCs) with 95% confidence intervals (CIs) were used to evaluate intra-observer and inter-observer variability. All statistical analyses were performed with JMP version 10.0 software (SAS Institute Inc., Cary, North Carolina).

This study was approved by the Mayo Clinic Institutional Review Board.

## Results

### Baseline characteristics

Baseline characteristics are presented in Table 1. Of the 106 patients screened, four patients were excluded due to suboptimal image quality. Full-thickness strain analysis was performed in the remaining 102 patients in the cohort of whom 63 (62%) were female with a mean age of 39 ± 15 years. Among the 20 patients who underwent further layer-specific strain analysis, 10 (50%) were female with a median age of 36 ± 13 years.

**Table 1:** Baseline characteristics (n = 102)

Characteristics	Full-Thickness Analysis
Clinical	
Age (years)	39 ± 15
Female	63 (62%)
Body mass index (kg/m^2^)	25.8 ± 5.0
Heart rate (bpm)	73 ± 14
Systolic blood pressure (mmHg)	115 ± 15
Diastolic blood pressure (mmHg)	71 ± 10
Echocardiographic	
LV end-diastolic diameter (mm)	47 ± 4
LV end-systolic diameter (mm)	30 ± 3
LV ejection fraction (%)	64 ± 4
E velocity (m/s)	0.8 ± 0.2
A velocity (m/s)	0.5 ± 0.1
Septal e’ velocity (m/s)	0.11 ± 0.03
E/A ratio	1.6 ± 0.6
Septal E/e′ ratio	7.6 ± 1.9
RV systolic pressure (mmHg)	25 ± 4

*Abbreviations*: *A* Late mitral inflow velocity, *E* Early mitral inflow velocity, *e’* Mitral annulus tissue velocity, *LV* Left ventricular, *RV* Right ventricular

The proportion of female subjects is expressed as number (%). Continuous data are expressed as mean ± standard deviation

### Left ventricular full-thickness strain

Table 2 displays the mean full-thickness LV global longitudinal, circumferential, and radial strain, systolic strain rate, early diastolic strain rate, and respective time-to-peak measurements for patients overall and when stratified by age and sex. Mean LV global longitudinal, circumferential, and radial strain measured − 18.4 ± 2.0%, − 22.1 ± 4.1%, and 43.9 ± 12.1%, respectively. Women displayed higher mean LV longitudinal and circumferential systolic strain and longer mean LV longitudinal, circumferential, and radial time-to-peak strain compared with men. There was no consistent association between age and the various strain measurements, with LV longitudinal early diastolic strain rate being the only parameter that showed a statistically significant decrease with increasing age.

**Table 2:** Full-thickness left ventricular strain analysis

Variable	All subjects (n = 102)	Men (n = 39)	Women (n = 63)	*P* Value
Longitudinal				
S (%)	−18.4 ± 2.0	− 17.4 ± 1.5	− 19.0 ± 2.0	< 0.001
SRs (1/s)	−0.99 ± 0.12	−0.95 ± 0.09	−1.02 ± 0.12	0.004
SRe (1/s)	1.06 ± 0.20	0.99 ± 0.15	1.10 ± 0.21	0.008
S-TP (ms)	378.7 ± 43.4	367.7 ± 40.9	385.7 ± 43.8	0.04
SRs-TP (ms)	196.3 ± 30.7	189.9 ± 33.5	200.4 ± 28.2	0.09
SRe-TP (ms)	502.9 ± 50.5	492.6 ± 48.8	509.4 ± 50.8	0.11
Circumferential				
S (%)	−22.1 ± 4.1	−20.5 ± 3.9	− 23.1 ± 3.9	0.002
SRs (1/s)	−1.40 ± 0.29	−1.37 ± 0.29	−1.42 ± 0.30	0.35
SRe (1/s)	1.39 ± 0.35	1.26 ± 0.33	1.47 ± 0.34	0.003
S-TP (ms)	374.4 ± 47.4	360.9 ± 43.0	383.0 ± 48.3	0.02
SRs-TP (ms)	217.0 ± 38.4	210.2 ± 34.0	221.4 ± 40.6	0.16
SRe-TP (ms)	498.8 ± 54.4	491.1 ± 47.8	503.7 ± 58.1	0.26
Radial				
S (%)	43.9 ± 12.1	43.7 ± 11.5	44.0 ± 12.6	0.89
SRs (1/s)	2.17 ± 0.62	2.17 ± 0.64	2.17 ± 0.62	0.98
SRe (1/s)	−2.07 ± 0.73	−1.91 ± 0.65	−2.17 ± 0.76	0.09
S-TP (ms)	381.1 ± 66.1	364.3 ± 62.7	391.9 ± 66.5	0.04
SRs-TP (ms)	204.1 ± 41.7	192.4 ± 33.7	211.6 ± 44.7	0.02
SRe-TP (ms)	507.2 ± 62.9	502.3 ± 61.7	510.3 ± 64.0	0.54

*Abbreviations: S* Systolic strain, *SRe* Early diastolic strain rate, *S-TP* Time-to-peak strain, *SRe-TP* Time-to-peak early diastolic strain rate, *SRs* Systolic strain rate, *SRs-TP* Time-to-peak systolic strain rate

Continuous data are expressed as mean ± standard deviation

### Right ventricular strain

Mean RV global and free wall longitudinal systolic strain, systolic strain rate, and early diastolic strain rate are outlined in Table 3. Mean RV global and free wall longitudinal systolic strain measured − 24.2 ± 3.9% and − 26.1 ± 5.2%, respectively. As with LV strain, mean RV global and free wall longitudinal systolic strain, systolic strain rate, and early diastolic strain rate were higher in women than in men and there was no consistent association with age.

**Table 3:** Full-thickness right ventricular strain analysis

Variable	All subjects (n = 102)	Men (n = 39)	Women (n = 63)	*P* Value
Longitudinal (global)				
S (%)	−24.2 ± 3.9	− 22.3 ± 3.4	−25.4 ± 3.8	0.001
SRs (1/s)	−1.4 ± 0.3	−1.3 ± 0.3	−1.5 ± 0.3	0.01
SRe (1/s)	1.5 ± 0.4	1.3 ± 0.3	1.6 ± 0.5	0.001
S-TP (ms)	389.8 ± 53.0	379.5 ± 54.3	396.4 ± 51.5	0.12
SRs-TP (ms)	213.7 ± 41.4	205.7 ± 38.4	218.8 ± 42.7	0.13
SRe-TP (ms)	510.4 ± 58.7	499.7 ± 53.5	517.2 ± 61.2	0.15
Longitudinal (free wall)				
S (%)	−26.1 ± 5.2	−24.5 ± 4.4	−27.1 ± 5.4	0.01
SRs (1/s)	−1.6 ± 0.4	−1.5 ± 0.3	−1.6 ± 0.5	0.07
SRe (1/s)	1.9 ± 0.8	1.6 ± 0.8	2.0 ± 0.8	0.01
S-TP (ms)	395.5 ± 78.2	384.7 ± 64.6	404.4 ± 72.8	0.25
SRs-TP (ms)	229.5 ± 61.3	224.6 ± 60.3	232.7 ± 62.2	0.52
SRe-TP (ms)	510.32 ± 75.9	502.1 ± 80.7	515.6 ± 72.8	0.41

*Abbreviations: S* Systolic strain, *SRe* Early diastolic strain rate, *S-TP* Time-to-peak strain, *SRe-TP* Time-to-peak early diastolic strain rate, *SRs* Systolic strain rate, *SRs-TP* Time-to-peak systolic strain rate

Continuous data are expressed as mean ± standard deviation

### Left ventricular layer-specific strain

Table 4 demonstrates mean LV global systolic strain, strain rate, and respective time-to-peak values depending on the layer analyzed for the 20 patients in whom layer-specific strain analysis was performed. Mean longitudinal, circumferential, and radial systolic strain, systolic strain rate, and early diastolic strain rate were lower in the epicardial-only layer compared with the full-thickness and endocardial-only layers (*P* ≤ 0.02). There was no significant difference in strain measurements between the full-thickness and endocardial-only layers.

**Table 4:** Layer-specific global left ventricular strain analysis (n = 20)

Variable	Full-thickness	Endocardial-only	Epicardial-only	*P* Value	Intra-class correlation coefficient (95% confidence interval)
Longitudinal					
S (%)	−19.2 ± 2.2	− 19.1 ± 2.1	−16.5 ± 1.6^a^	< 0.001	0.97 (0.71–0.94)
SRs (1/s)	−1.0 ± 0.1	−1.1 ± 0.1	−0.9 ± 0.1^a^	< 0.001	0.97 (0.76–0.95)
SRe (1/s)	1.1 ± 0.3	1.1 ± 0.3	0.9 ± 0.2^a^	< 0.001	0.97 (0.85–0.97)
S-TP (ms)	380.1 ± 42.6	381.8 ± 43.4	373.5 ± 41.5	0.22	0.97 (0.84–0.99)
SRs-TP (ms)	197.5 ± 29.3	201.4 ± 32.6	194.7 ± 32.0	0.59	0.91 (0.83–0.96)
SRe-TP (ms)	499.4 ± 46.6	500.2 ± 45.5	496.9 ± 47.7	0.86	0.98 (0.96–0.99)
Circumferential					
S (%)	−23.6 ± 3.9	−23.7 ± 3.9	− 16.7 ± 4.2^a^	< 0.001	0.96 (0.50–0.89)
SRs (1/s)	− 1.5 ± 0.3	− 1.5 ± 0.3	− 1.1 ± 0.3^a^	< 0.001	0.96 (0.71–0.94)
SRe (1/s)	1.5 ± 0.3	1.4 ± 0.3	1.0 ± 0.3^a^	< 0.001	0.94 (0.49–0.89)
S-TP (ms)	367.3 ± 40.4	365.8 ± 41.6	381.5 ± 44.5	0.44	0.71 (0.43–0.87)
SRs-TP (ms)	223.4 ± 32.2	220.6 ± 32.4	219.7 ± 43.3	0.97	0.74 (0.52–0.89)
SRe-TP (ms)	486.2 ± 41.1	483.0 ± 40.0	497.3 ± 55.0	0.57	0.73 (0.48–0.88)
Radial					
S (%)	39.8 ± 10.8	50.9 ± 22.9^b^	33.9 ± 11.1^c^	< 0.001	0.69 (0.18–0.82)
SRs (1/s)	1.8 ± 0.5	2.2 ± 0.7^b^	1.9 ± 0.7	0.02	0.71 (0.40–0.90)
SRe (1/s)	−1.7 ± 0.7	− 2.4 ± 1.5^b^	−1.6 ± 0.8	0.01	0.60 (0.08–0.80)
S-TP (ms)	382.6 ± 46.9	414.0 ± 70.6	408.7 ± 58.5	0.18	0.66 (0.30–0.84)
SRs-TP (ms)	212.1 ± 53.0	201.4 ± 62.7	202.3 ± 47.0	0.85	0.52 (0.19–0.82)
SRe-TP (ms)	501.1 ± 47.6	491.3 ± 54.7	510.8 ± 58.1	0.34	0.81 (0.63–0.92)

*Abbreviations: S* Systolic strain, *SRe* Early diastolic strain rate, *S-TP* Time-to-peak strain, *SRe-TP* Time-to-peak early diastolic strain rate, *SRs* Systolic strain rate, *SRs-TP* Time-to-peak systolic strain rate

Continuous data are expressed as mean ± standard deviation

^a^*P <* 0.001 when compared with full-thickness and endocardial-only groups

^b^*P* < 0.05 when compared with full-thickness group

^c^*P <* 0.01 when compared with full-thickness and endocardial-only groups

### Left ventricular segmental strain

Mean segmental LV longitudinal and circumferential strain parameters corresponding to the different layers are presented in Table 5. Similar to the global strain analysis, segmental longitudinal and circumferential strain measurements were lowest in the epicardium compared with the full-thickness and endocardial-only layers (*P* < 0.001), which showed no significant difference between them (*P* > 0.05). In contrast, the segmental radial strain parameters demonstrated poor intra- and inter-observer variability based on the suboptimal intra-class correlation coefficients and are therefore not presented in Table 5 but available for review in Supplementary Table 2.

**Table 5:** Layer-specific segmental left ventricular longitudinal and circumferential strain analysis (n = 20)

Variable	Full-thickness	Endocardial-only	Epicardial-only	*P* Value	Intra-class correlation coefficient(95% confidence interval)
Segmental longitudinal S (%)					
Anterior	−19.8 ± 3.1	−19.6 ± 3.1	−17.1 ± 2.8^a^	< 0.001	0.96 (0.82–0.96)
Anteroseptal	− 19.1 ± 3.1	−19.1 ± 2.9	−16.5 ± 2.4^a^	< 0.001	0.90 (0.68–0.93)
Inferior	− 19.8 ± 3.2	−19.7 ± 3.3	−17.0 ± 3.0^a^	< 0.001	0.94 (0.78–0.95)
Lateral	− 19.8 ± 3.0	−19.6 ± 2.9	−15.9 ± 3.2^a^	< 0.001	0.94 (0.65–0.92)
Posterior	−18.2 ± 3.1	− 18.2 ± 3.2	−15.0 ± 1.9^a^	< 0.001	0.94 (0.70–0.93)
Septal	− 19.1 ± 2.6	− 19.0 ± 2.3	− 16.2 ± 2.5^a^	< 0.001	0.89 (0.58–0.91)
Segmental longitudinal SRs (1/s)					
Anterior	− 1.1 ± 0.2	− 1.1 ± 0.2	− 0.9 ± 0.2^a^	< 0.001	0.94 (0.80–0.96)
Anteroseptal	− 1.1 ± 0.2	− 1.1 ± 0.2	−1.0 ± 0.2^a^	< 0.001	0.92 (0.77–0.94)
Inferior	−1.1 ± 0.2	−1.1 ± 0.2	− 0.9 ± 0.2^a^	< 0.001	0.91 (0.74–0.94)
Lateral	− 1.1 ± 0.2	−1.1 ± 0.2	− 0.9 ± 0.2^a^	< 0.001	0.94 (0.68–0.93)
Posterior	− 1.1 ± 0.2	−1.1 ± 0.2	−0.9 ± 0.2^a^	< 0.001	0.93 (0.67–0.93)
Septal	− 1.1 ± 0.2	−1.1 ± 0.1	− 0.9 ± 0.2^a^	< 0.001	0.84 (0.56–0.90)
Segmental circumferential S (%)					
Anterior	−24.6 ± 7.0	−24.7 ± 6.7	− 15.3 ± 5.0^a^	< 0.001	0.92 (0.53–0.90)
Anteroseptal	− 24.9 ± 6.0	− 25.2 ± 6.4	− 16.9 ± 5.2^a^	< 0.001	0.94 (0.60–0.91)
Inferior	− 23.7 ± 5.9	− 23.7 ± 6.4	− 18.8 ± 6.8^a^	< 0.001	0.90 (0.73–0.94)
Lateral	− 22.7 ± 6.6	−22.7 ± 6.4	− 15.2 ± 5.2^a^	< 0.001	0.92 (0.63–0.92)
Posterior	− 23.9 ± 7.4	− 23.5 ± 7.5	− 16.4 ± 4.9^a^	< 0.001	0.92 (0.66–0.93)
Septal	−22.0 ± 5.8	−22.3 ± 5.7	− 17.7 ± 6.5^a^	< 0.001	0.84 (0.59–0.91)
Segmental circumferential SRs (1/s)					
Anterior	− 1.5 ± 0.5	− 1.5 ± 0.5	−1.0 ± 0.4^a^	< 0.001	0.86 (0.54–0.90)
Anteroseptal	−1.6 ± 0.5	−1.6 ± 0.5	−1.1 ± 0.5^a^	< 0.001	0.95 (0.75–0.94)
Inferior	−1.6 ± 0.5	−1.6 ± 0.5	−1.2 ± 0.5^a^	< 0.001	0.95 (0.82–0.96)
Lateral	−1.4 ± 0.4	−1.4 ± 0.4	−1.0 ± 0.4^a^	< 0.001	0.83 (0.50–0.89)
Posterior, 1/s	−1.5 ± 0.6	−1.5 ± 0.6	−1.1 ± 0.5^a^	< 0.001	0.96 (0.82–0.96)
Septal, 1/s	−1.4 ± 0.5	−1.4 ± 0.4	−1.2 ± 0.5^b^	0.009	0.86 (0.70–0.93)

*Abbreviations: S* Systolic strain, *SRs* Systolic strain rate

Continuous data are expressed as mean ± standard deviation

^a^*P* < 0.001 when compared with full-thickness and endocardial-only groups

^b^*P <* 0.05 when compared with full-thickness and endocardial-only groups

### Left ventricular strain by ventricular level

Longitudinal systolic strain and systolic strain rate were highest in the apex compared with the mid and basal LV in the endocardial-only and full-thickness layers (*P* < 0.001) but not in the epicardial-only layer (Table 6). There was no statistically significant difference in layer-specific longitudinal systolic strain and systolic strain rate between the mid and basal LV.

**Table 6:** Layer-specific left ventricular longitudinal strain analysis by ventricular level (n = 20)

Variable	Full-thickness	Endocardial-only	Epicardial-only	*P* Value	Intra-class correlation coefficient(95% confidence interval)
Level-specific longitudinal S (%)					
Basal	−18.1 ± 2.1	−18.2 ± 2.0	−16.4 ± 2.0^a^	< 0.001	0.90 (0.68–0.93)
Mid	− 18.2 ± 2.4	−18.1 ± 2.4	− 16.7 ± 2.1^b^	< 0.001	0.96 (0.86–0.97)
Apical	− 21.6 ± 3.1^c^	− 21.3 ± 3.2^c^	− 16.8 ± 2.1^b^	< 0.001	0.91 (0.23–0.83)
*P* value	< 0.001	< 0.001	0.09		
Level-specific longitudinal SRs (1/s)					
Basal	−1.01 ± 0.16	− 1.03 ± 0.15	−0.97 ± 0.16^a^	0.06	0.92 (0.82–0.96)
Mid	−0.99 ± 0.14	− 1.00 ± 0.14	−0.91 ± 0.11^a^	< 0.001	0.96 (0.86–0.97)
Apical	− 1.21 ± 0.26^c^	−1.20 ± 0.16^c^	−0.95 ± 0.16^b^	< 0.001	0.87 (− 0.11–0.75)
*P* value	< 0.001	< 0.001	0.07		

*Abbreviations: S* Systolic strain, *SRs* Systolic strain rate

Continuous data are expressed as mean ± standard deviation

^a^*P* < 0.005 when compared with full-thickness and endocardial-only

^b^*P* < 0.001 when compared with full-thickness and endocardial-only groups

^c^*P* < 0.001 when compared with basal and mid left ventricular levels

### Reproducibility analysis

The intra-observer variability and inter-observer variability for the various global full-thickness strain measurements are presented in Table 7. Intra-class correlation coefficients were the highest for global longitudinal strain measurements, followed closely by circumferential strain measurements, and the lowest for global radial strain measurements.

**Table 7:** Intra- and inter-observer variability of global full-thickness left ventricular strain

	Intra-class correlation coefficient(95% confidence interval)	
Variable	Intra-observer variability	Inter-observer variability
Longitudinal		
S	0.998 (0.994–0.999)	0.996 (0.989–0.998)
SRs	0.995 (0.988–0.998)	0.982 (0.956–0.993)
SRe	0.964 (0.913–0.986)	0.994 (0.984–0.997)
Circumferential		
S	0.997 (0.994–0.999)	0.993 (0.983–0.997)
SRs	0.994 (0.984–0.997)	0.975 (0.940–0.990)
SRe	0.967 (0.919–0.987)	0.967 (0.919–0.987)
Radial		
S	0.857 (0.751–0.933)	0.845 (0.727–0.927)
SRs	0.906 (0.782–0.961)	0.898 (0.818–0.953)
SRe	0.808 (0.668–0.909)	0.871 (0.477–0.918)

*Abbreviations: S* Systolic strain, *SRe* Early diastolic strain rate, *SRs* Systolic strain rate

## Discussion

We have presented an analysis of ventricular strain performed within a real world cohort of patients with no structural heart disease using a vendor-independent speckle-tracking software. We measured longitudinal, circumferential, and radial systolic strain, strain rate, and respective time-to-peak values both globally and based on several parameters including age, sex, wall segment, ventricular level, and myocardial layer, while providing information on the reproducibility of these measurements. Full-thickness mean LV global longitudinal, circumferential, and radial systolic strain measured − 18.4 ± 2.0%, − 22.1 ± 4.1%, and 43.9 ± 12.1% respectively, while mean RV global and free wall longitudinal systolic strain measured − 24.2 ± 3.9% and − 26.1 ± 5.2% respectively.

Our results were generally similar to those found in previous studies with some differences. In a meta-analysis of 2597 healthy subjects from 24 studies, average values of LV global longitudinal, circumferential, and radial strain overlapped with our findings and were reported to be − 19.7% (95% CI − 20.4% to − 18.9%), − 23.3% (95% CI − 24.6% to − 22.1%), and 47.3% (95% CI 43.6% to 51.0%) [21]. Differences in characteristics of patients included in the meta-analysis as well as differences in vendor software used for the strain analyses could account for the corresponding differences in results [27–31].

On the other hand, in a study of 549 healthy subjects enrolled in 22 European institutions in whom strain was measured using the same vendor-independent 2D Cardiac Performance Analysis software by TomTec, LV global longitudinal strain was similar at − 22.5 ± 2.7% while absolute global circumferential and radial strain values were much higher at − 31.9 ± 4.5, and 37.4 ± 8.4% respectively compared to our study [18]. This could be related to the differences in method used to measure circumferential and radial strain. While we analyzed strain at the level of the papillary muscle, that study also included the basal and apical levels for analysis as well.

The results of our analysis confirm a number of characteristics of ventricular systolic strain that have been shown previously in separate studies but have infrequently been demonstrated within the same cohort, as we have done in this study. First, absolute values of the various ventricular strain parameters are higher in women than in men [7, 11, 13, 16–18] and in the RV compared with the LV [12]. Furthermore, systolic strain parameters are higher in the endocardium than in the epicardium and in apex compared with the base when measured in the endocardial layer [7, 9, 10, 14, 16, 17]. Compared to the base, the apex is smaller and is subject to less wall stress, which may result in relatively higher global longitudinal strain [9]. In addition, global longitudinal systolic strain is the most reproducible measure of strain, followed closely by global circumferential systolic strain, whereas global radial systolic strain demonstrates suboptimal intra- and inter-observer variability [12]. Radial strain may be less reproducible than longitudinal strain since the spread of myocardium is smaller in this direction resulting in fewer speckles to track and therefore making them more vulnerable to the impact of uncertain boundaries. Furthermore, unlike longitudinal strain, circumferential and radial strain represents myocardial fiber displacement that is not occurring parallel to the ultrasound beam [32].

The impact of age on strain measurements is less clear. In our study, there was no statistically significant difference in full-thickness LV or RV systolic strain or systolic strain rate between the three age categories examined. Statistical significance was only found in the LV early diastolic strain rate and RV time-to-peak systolic strain rate, which given the isolated nature of these findings, have unclear clinical significance. These findings may be due to our small sample size. Nevertheless, prior studies have also demonstrated conflicting results regarding the influence of age on strain, with one study showing no significant correlation [33] while several other studies suggested a decline in LV or RV longitudinal or radial systolic strain and systolic strain rate with age [11, 12, 34].

More recent studies suggest that there may be a more complex interaction between age and strain, depending on the type of strain as well as the level and layer within the ventricle [7, 17, 18]. Longitudinal systolic strain appears to generally decrease with age whereas circumferential and radial systolic strain measurements appear to increase [17, 18]. However, longitudinal systolic strain does not appear to uniformly decrease within the ventricle and instead decreases more in the base and increases more in the apex with age [7, 17]. Regional variability in strain and differences in the mean age of subjects in a given cohort could partly account for differences in age-related changes in strain observed between studies. Of note, in the meta-analysis of 24 studies that reported normal ranges of LV systolic strain, only two included subjects with a mean age of ≥65 years [21]. Given the increasing proportion of patients aged ≥65 years referred for echocardiography, more precise characterization of strain in this population would be a worthwhile area of further investigation.

Similarly, use of the vendor-independent 2D Cardiac Performance Analysis software (TomTec Imaging System, Munich, Germany) is under-represented in studies that report normal reference ranges for strain despite its increasing use in clinical practice and research. Among the 28 data sets included in the meta-analysis outlining normal ranges of LV strain, 23 of them used EchoPAC software (GE Healthcare, Milwaukee, WI) and none of the remaining 5 data sets used the 2D Cardiac Performance Analysis software for strain analysis [21]. To date, only one other study has presented normal ranges of LV strain using this software and these patients were healthy subjects referred to various institutions in Europe [18]. Our study not only increases the representation of this software in reporting reference ranges of LV strain but also provides measurements of systolic and early diastolic strain rates, respective time-to-peak values, layer-specific strain, and RV longitudinal global and free wall systolic strain in a North American cohort, which has not been described previously.

### Limitations

The present study must be interpreted with the following limitations in mind. The sample size of our study was small. Strain analysis was performed retrospectively. Patients in our cohort had a clinically justified reason to be referred for echocardiography, which may introduce selection bias. We could not fully exclude patients with non-cardiac comorbidities that could have potentially influenced myocardial deformation in some way. Our images were acquired using the Vivid 7 GE ultrasound system and analyzed using TomTec Imaging Systems, which could limit the generalizability of the findings. Furthermore, our strain measurements could have been slightly different if another definition of end-diastole or end-systole were chosen. However, such differences would likely be negligible in our cohort of patients with no regional wall motion abnormalities. In addition, the speckle-tracking software used in this study follows an algorithm that relies predominantly on the inner boundary traced even when both an inner and outer boundary are provided, which explains the similar strain measurements between the full-thickness and endocardial-only layers. As well, for our analysis of layer-specific strain, the mid-myocardial layer was not assessed individually as it was difficult to isolate the mid-myocardial layer without significant overlap with the endocardial and epicardial layers. Nevertheless, a prior study demonstrated good agreement in global longitudinal strain by default vendor layer in the mid myocardium for the GE platform and the endocardium for TomTec [35]. Finally, concomitant measurements of ventricular strain using other modalities such as magnetic resonance imaging or sonomicrometry were not available for comparison as this was beyond the scope of our study. Instead, we aimed to demonstrate the expected range of ventricular strain measured by 2D speckle-tracking echocardiography in patients without structural heart disease.

## Conclusions

We performed an analysis of ventricular strain in a cohort of patients with no evidence of structural heart disease using the vendor-independent 2D Cardiac Performance Analysis software by TomTec. Women demonstrate higher absolute values of strain than men. Longitudinal strain is the most reproducible measure of strain while radial strain is the least reproducible. Strain is highest at the apex and in the endocardium. Further study is warranted to clarify the role of age on strain, particularly in elderly individuals who represent the majority of patients referred for cardiovascular evaluation. Our study provides reference ranges for various parameters of strain that may be encountered in a real world cohort of patients referred for echocardiography.

## Supplementary information

**Additional file 1: Supplementary Table 1.** Indications for echocardiography

**Additional file 2: Supplementary Table 2.** Layer-specific left ventricular segmental radial strain analysis (n = 20)

## Data Availability

Public access to the database is closed. All requests for raw and analyzed data and related materials will be reviewed by the Mayo Clinic legal department to verify whether the request is subject to any intellectual property or confidentiality obligations. Requests for patient-related data not included in the paper will not be considered. Any data and materials that can be shared will be released via a Material Transfer Agreement.

## Abbreviations

2D: 2-dimensional
A: Late mitral inflow velocity
E: Early mitral inflow velocity
e’: Mitral annulus tissue velocity
EF: Ejection fraction
ICC: Intra-class correlation coefficient
LV: Left ventricle
RV: Right ventricle
S: Systolic strain
S-TP: Time-to-peak strain
SRe: Early diastolic strain rate
SRe-TP: Time-to-peak early diastolic strain rate
SRs: Systolic strain rate
SRs-TP: Systolic strain rate
