# Supplementary material for: Ventricular strain analysis in patients with no structural heart disease using a vendor-independent speckle-tracking software
Source: BMC Cardiovasc Disord. 2020 Jun 5;20:274. doi: 10.1186/s12872-020-01559-1 (PMC7275339; doi:10.1186/s12872-020-01559-1)
Supplement: Supplementary file 1 — Additional file 1: Supplementary Table 1. Indications for echocardiography [file 12872_2020_1559_MOESM1_ESM.docx]

| **Supplementary Table 1** Indications for echocardiography | | |
| --- | --- | --- |
| Indication group | Indication | *n* |
| Group 1 | Baseline evaluation prior to chemotherapy | 28 |
|  | Cardiac symptoms: | 9 |
|  | Palpitations | 4 |
|  | Chest pain | 3 |
|  | Dyspnea | 1 |
|  | Syncope | 1 |
|  | Murmur on physical examination | 1 |
|  | Abnormal electrocardiogram | 4 |
| Group 2 | Family history of cardiomyopathy: | 24 |
|  | Hypertrophic cardiomyopathy | 17 |
|  | Idiopathic dilated cardiomyopathy | 3 |
|  | Amyloid cardiomyopathy | 3 |
|  | Non-compaction cardiomyopathy | 1 |
| Group 3 | Systemic illness predisposing to cardiomyopathy: | 14 |
|  | Amyloidosis | 12 |
|  | Sarcoidosis | 1 |
|  | Hypereosinophilia | 1 |
|  | Muscular dystrophy | 3 |
|  | Suspected but unconfirmed mitochondrial disorder | 2 |
|  | Prior radiation therapy | 3 |
|  | Prior chemotherapy | 15 |
|  | Anthracyclines | 11 |
|  | Trastuzumab | 2 |
|  | Other chemotherapy | 3 |
