# Supplementary material for: Ventricular strain analysis in patients with no structural heart disease using a vendor-independent speckle-tracking software
Source: BMC Cardiovasc Disord. 2020 Jun 5;20:274. doi: 10.1186/s12872-020-01559-1 (PMC7275339; doi:10.1186/s12872-020-01559-1)
Supplement: Supplementary file 2 — Additional file 2: Supplementary Table 2. Layer-specific left ventricular segmental radial strain analysis (n = 20) [file 12872_2020_1559_MOESM2_ESM.docx]

**Supplementary Table 2** Layer-specific left ventricular segmental radial strain analysis (n=20)

| Variable | Full-thickness | Endocardial-only | Epicardial-only | *P* Value | Intra-class correlation coefficient  (95% confidence interval) |
| --- | --- | --- | --- | --- | --- |
| Segmental radial S (%) |  |  |  |  |  |
| Anterior | 35.7 ± 13.9 | 53.7 ± 42.8^a^ | 37.5 ± 15.4 | 0.06 | 0.65 (0.25-0.83) |
| Anteroseptal | 32.3 ± 14.9 | 39.6 ± 23.9 | 27.2 ± 14.9 | 0.07 | 0.63 (0.21-0.82) |
| Inferior | 49.1 ± 25.8 | 76.7 ± 30.5^b^ | 31.1 ± 19.3^c^ | 0.01 | 0.63 (0.15-0.81) |
| Lateral | 36.7 ± 18.9 | 42.0 ± 39.2 | 48.6 ± 22.1 | 0.19 | 0.79 (0.58-0.91) |
| Posterior | 46.2 ± 24.7 | 49.8 ± 29.3 | 32.7 ± 16.7^c^ | 0.004 | 0.84 (0.63-0.92) |
| Septal | 38.8 ± 14.6 | 43.8 ± 32.4 | 25.9 ± 14.3^c^ | 0.07 | 0.49 (−0.19-0.74) |
| Segmental radial SRs (1/s) |  |  |  |  |  |
| Anterior | 1.75 ± 0.46 | 2.04 ± 0.91 | 1.99 ± 0.82 | 0.55 | 0.42 (−0.12-0.75) |
| Anteroseptal | 1.49 ± 0.52 | 1.87 ± 0.89 | 1.71 ± 0.78 | 0.31 | 0.49 (−0.02-0.77) |
| Inferior | 2.01 ± 0.94 | 2.80 ± 1.71^a^ | 1.71 ± 1.03^c^ | 0.001 | 0.79 (0.47-0.88) |
| Lateral | 1.82 ± 1.00 | 1.93 ± 1.09 | 2.44 ± 1.24^a^ | 0.06 | 0.80 (0.58-0.91) |
| Posterior | 2.07 ± 1.27 | 2.34 ± 1.47 | 2.00 ± 1.17 | 0.47 | 0.88 (0.76-0.95) |
| Septal | 1.85 ± 0.77 | 2.19 ± 1.29 | 1.48 ± 0.65^c^ | 0.01 | 0.73 (0.40-0.87) |
|  |  |  |  |  |  |

*Abbreviations:* S = systolic strain, SRs = systolic strain rate.

Continuous data are expressed as mean ± standard deviation.

^a^ *P*<0.05 when compared to full-thickness group.

^b^ *P*<0.001 when compared to full-thickness group.

^c^ *P<*0.05 when compared with full-thickness and endocardial-only groups.
